# A new technique for testing distribution of knowledge and to estimate sampling sufficiency in ethnobiology studies

**DOI:** 10.1186/1746-4269-8-11

**Published:** 2012-03-15

**Authors:** Thiago Antonio Sousa Araújo, Alyson Luiz Santos Almeida, Joabe Gomes Melo, Maria Franco Trindade Medeiros, Marcelo Alves Ramos, Rafael Ricardo Vasconcelos Silva, Cecília Fátima Castelo Branco Rangel Almeida, Ulysses Paulino Albuquerque

**Affiliations:** 1 Departamento de Biologia, Área Botânica, Laboratório de Etnobotânica Aplicada, Universidade Federal Rural de Pernambuco, Rua Dom Manoel de Medeiros s/n, Dois Irmãos, Recife, Pernambuco 52171-030, Brazil; 2 Centro de Ensino Superior do Vale do São Francisco, Rua Cel. Trapiá, Centro, Belém, de São Francisco, Pernambuco 56440-000, Brazil

**Keywords:** quantitative indices, selection of informants, outliers, intentional samplings

## Abstract

**Background:**

We propose a new quantitative measure that enables the researcher to make decisions and test hypotheses about the distribution of knowledge in a community and estimate the richness and sharing of information among informants. In our study, this measure has two levels of analysis: intracultural and intrafamily.

**Methods:**

Using data collected in northeastern Brazil, we evaluated how these new estimators of richness and sharing behave for different categories of use.

**Results:**

We observed trends in the distribution of the characteristics of informants. We were also able to evaluate how outliers interfere with these analyses and how other analyses may be conducted using these indices, such as determining the distance between the knowledge of a community and that of experts, as well as exhibiting the importance of these individuals' communal information of biological resources. One of the primary applications of these indices is to supply the researcher with an objective tool to evaluate the scope and behavior of the collected data.

## Introduction

Over the past several years, especially since the 1990s, many techniques for the quantitative analysis of traditional botanical knowledge have been proposed. Perhaps some of the most popular techniques are the use value proposed by Phillips and Gentry [1,2] and the relative importance proposed by Bennett and Prance [3]. In general, these proposals are within the scope of a set of techniques (referred to as the "consensus of informants") aimed at assessing the relative importance of a given resource using the consensus of the informants' responses. A set of techniques that is less popular but has long been the subject of discussion was proposed to assess the so-called "cultural importance" of a resource (plants, for example); this set was labeled in its generality as "subjective allocation techniques" [4,5]. The subjective allocation techniques have been harshly criticized because the researcher must compute, according to his vision, priority scores in order to assign importance to a resource. Today, over 80 different techniques incorporate computations that appear to have the same goals. Medeiros et al. [6] as certain that many new technical proposals do not actually introduce new features but serve only to inflate the literature; their creation is unnecessary, due to their redundancy. A general analysis shows that the vast majority of these indices are intended to assign importance to a given biological resource

We analyze the degree to which people from the same family nucleus or unit share knowledge about useful species as a proxy for this investigation. Numerous ethnobotanical studies have proposed quantitative analyses based on the "Consensus of Informants", i.e., the degree to which the informants of a community share information on the use of specific resources e.g., [7,8]. For example, when opting to collect information only from one family member or from a sampling of the community, logistical constraints may prevent that researcher from collecting the entirety of the family or community's available information.

This can occur because there is a diversity of knowledge regarding biological resources see [8,9], and differences in knowledge may be a consequence of the role and activities that each social actor plays in their community or family, as well as the level of specialization needed to attain knowledge of certain features [8,10]. Sometimes, this makes it necessary to interview people at different positions in the social structure of a community until no new information is mentioned to the researcher [11]. Because the "knowledge can also be idiosyncratic, randomly distributed, shared within a subgroup, or contested by two or more different groups that 'know' different things" [12]. Hence,

1. People inside the same family nucleus or community may have considerably different knowledge of the richness of useful resources due to learning processes associated with their social position.

2. People inside the same family nucleus or community do not always communicate to other members about the use of a specific biological resource. Such communication will depend on several factors but is especially related to the uses of that resource (e.g., medicine, food, fuel and construction).

Depending on their research objectives and the financial and time resources available to execute them, ethnobiology studies can be oriented towards different types of informants. These informants may be chosen according to criteria such as age (e.g., children, adolescents and adults), gender, local recognition of expertise (i.e., people with increased knowledge about specific subjects) and generalist knowledge (i.e., people from the community or region who are not experts) and social roles (e.g., shamans and heads of families) when it is not possible or desirable to obtain the participation of the whole population. Commonly, researchers opt to conduct interviews with heads of families (one for each family unit e.g., [13-16]) to optimize the efficiency of their fieldwork based on the assumption, which is often not clearly explained by the researchers, that these individuals represent the knowledge of their family unit.

Modern ethnobiology studies face concerns of over sample sufficiency and, consequently, the representativeness of collected information [17,18]. Numerous researchers have attempted to solve these issues by importing tools from other disciplines, such as rarefaction curves and species-area curves from ecology [19-22]. These efforts attempt to estimate whether the sampling conducted in a study is sufficient to meet the representativeness criteria. Nevertheless, depending on how the authors use or interpret a specific procedure, the definition of the stability of a curve may be rendered arbitrary through subjective analysis. Peroni et al. [18] defend the use of ecological methods in ethnobotanical and ethnobiological investigations, stating that they enable "*evaluation of sampling effort, comparability among sets of data obtained in different regions, the possibility of objectively analyzing the distribution of ethnobotanical and ethnobiological knowledge, the possibility of applying ethnobotanical and ethnobiological studies for preservation programs, the possibility of integrating ethnobotanical and ethnobiological data and data of an ecological and biological character, and the possibility of evaluating biological and cultural standards*".

We propose a new quantitative measure that enables the researcher to make decisions and test hypotheses about the distribution of knowledge in a community and estimate the richness and sharing of information among informants. In our study, this measure has two levels of analysis: intracultural and intrafamily.

Although a broad range of quantitative indices are identified in the literature of ethnobiology, particularly indices that evaluate the consensus among informants regarding a biological resource, the creation of a new model was necessary due to the lack of simple techniques that can respond to issues raised in this study and the lack of indices that are able to evaluate the uniqueness of information. Our indices measure the consensus among people regarding their knowledge and its unique qualities. Two features make our approach distinctive; the first feature is the fact that the focus is placed on people and the knowledge that they possess. Another distinctive aspect is the sharing of information and the quality of uniqueness; indeed, uniqueness and the sharing of knowledge are rarely considered among the existing indices.

## Materials and methods

### Study area

**Figure 1:**
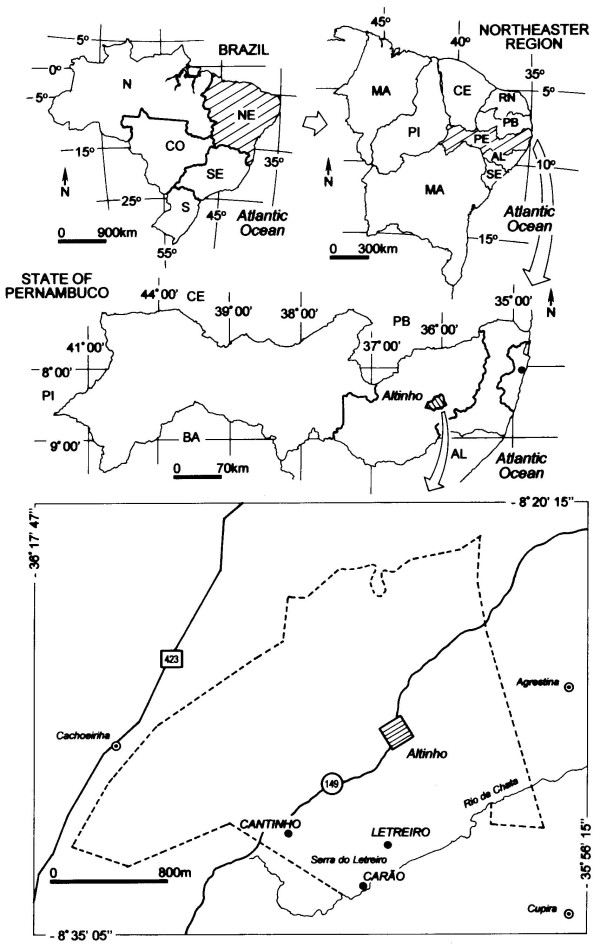
**Location of the community of Carão, Altinho municipality, Pernambuco state, Northeastern Brazil**. Source: ( 28).

This study was developed in the rural community of Carão (08°35'13.5″S, 36°05'34.6″W) located in the Altinho municipality, northeastern Brazil (Figure 1). The study community is situated 16 km from the center of the municipality. According to a survey that we conducted in the community health center in this area during the ethnobotanical survey, this community includes 189 inhabitants living in 61 houses, and 112 of them are older than 18 years of age (67 women and 45 men) [23]. The region has been the subject of previous ethnobiological investigations regarding the medicinal use of plants [23-25] and food plants [26,27], ecological hypotheses [28,29], landscape change [23,30], and the domestication and reproductive biology of native species [31,32]. Images of the study area are available at: https://picasaweb.google.com/etnobotanicaaplicada/CaraoAltinhoPernambucoNordesteBrasil

The community's economy is based on subsistence farming. The main crops are corn and beans, and some families breed animals such as chickens, goats and cattle to supplement their income. Both wild and cultivated vegetables supply daily family needs for food and health care. Moreover, individuals in the community use native and/or exotic plants for a number of purposes, including medicine, food, fuel, construction and fencing, foraging and veterinary medicine [24,29,32].

### Ethnobotanical Inventory

The objectives of this study were explained to both of the legal representatives of the municipality (the mayor and the secretary of agriculture and food supply) and at a monthly event open to the community to achieve representativeness from the public. We used this event to explain the procedures and aims of the research. Additionally, we requested permission to visit their homes. We conducted data collection in each house of the community. In this study, we are referring to each home as a family unit. In each house, we interviewed at least two people (usually members of a couple). Two brothers living in different houses were considered to be members of distinct families. From this data set, we composed two samples for analysis. The first sample consisted of an analysis of intrafamily plant knowledge, i.e., we sought to understand how knowledge is shared within the same family unit. For the second sample, we reorganized the data in order to perform an intracultural analysis. Thus, our two data sets were the family unit (intrafamily) and the community (intracultural).

Only people who were 18 years old or older were included among our sample informants. This age group was chosen because these individuals could legally account for their actions and using underage participants would require parental permission, which could make the study unfeasible. To access how discrepant data in a sample can influence the results, we performed our analyses considered the presence and absence of informants as outliers.

In addition, we analyzed the effect of outliers on data interpretation when their knowledge was compared to other community members. In this study, outliers are defined as informants with significant knowledge about a specific subject and/or category of resource use that distinguishes them from the group to which they belong.

With the support of community health agents, all residences were visited, and we explained the study to residents to confirm their participation. Confirmation was attained by having them sign an informed consent form (ICF) [24] according to ruling 196/96 of the National Health Council. This research was authorized by the ethics in research committee from Universidade Federal de Pernambuco, under number 238/06.

The survey of ethnobotanical data was conducted in three consecutive stages. First, a survey regarding knowledge and/or uses of plants for medicinal, food, and/or fuel purposes was conducted using the free-list technique [16]. Afterwards, a semi-structured interview was conducted [16] to record socioeconomic data. Finally, the guided tour technique was used [16] to enrich the list of cited plants and to collect vegetable material for incorporation into an herbarium. These plants were preserved and identified through comparisons with material in existing herbariums, consultations with experts, and references in specialized literature. Exsiccates were deposited at the Professor Vasconcelos Sobrinho Herbarium (PEUFR) in the Biology department of Pernambuco Federal Rural University.

### Preparation of data

A binary matrix was constructed that contained the record of citation for each species (Si) known to each informant (Ii) for each category of use (medicinal, food, or fuel).").

We focused only in three categories of use (medicinal, food, or fuel) because they are most common in the ethnobotany literature. Based on these data, the knowledge richness and uniqueness index (KRI) and the knowledge sharing index (KSI) were calculated. A description of how these indices were calculated is provided below.

### Mathematical Background

We used two quantitative measures, the knowledge richness index (KRI) and the knowledge sharing index (KSI), to calculate how the richness of N_s _known species are distributed and shared among N_i _informants inside the same family unit or throughout the community.

KRI measures the knowledge richness and uniqueness of a specific set of plants by a certain individual. The index tends to assume smaller values with a larger richness and a higher number of exclusive plants cited by a determined informant. KRI values are inversely proportional. In other words, a lower KRI value corresponds to the greater knowledge of the informant.

The KRI assumes values starting from zero and represents a distance measure that ranges from 0 to infinity. The more distant from zero the value presented by a determined informant, the smaller that the richness of a species known by that informant inside the family nucleus or group will be: $1/=\text{KRI}\Sigma {\text{J}}_{\text{i}}^{2}$ where: ${\text{J}}_{\text{i}}={\text{R}}_{\text{i}}/\text{R}{\text{f}}_{\text{i}}$

R_i _- Record of species (S_i_) cited by informant (I_i_);

Rf_i _- Total record of species (S_i_) cited by the family or community (f_i_).

**Figure 2:**
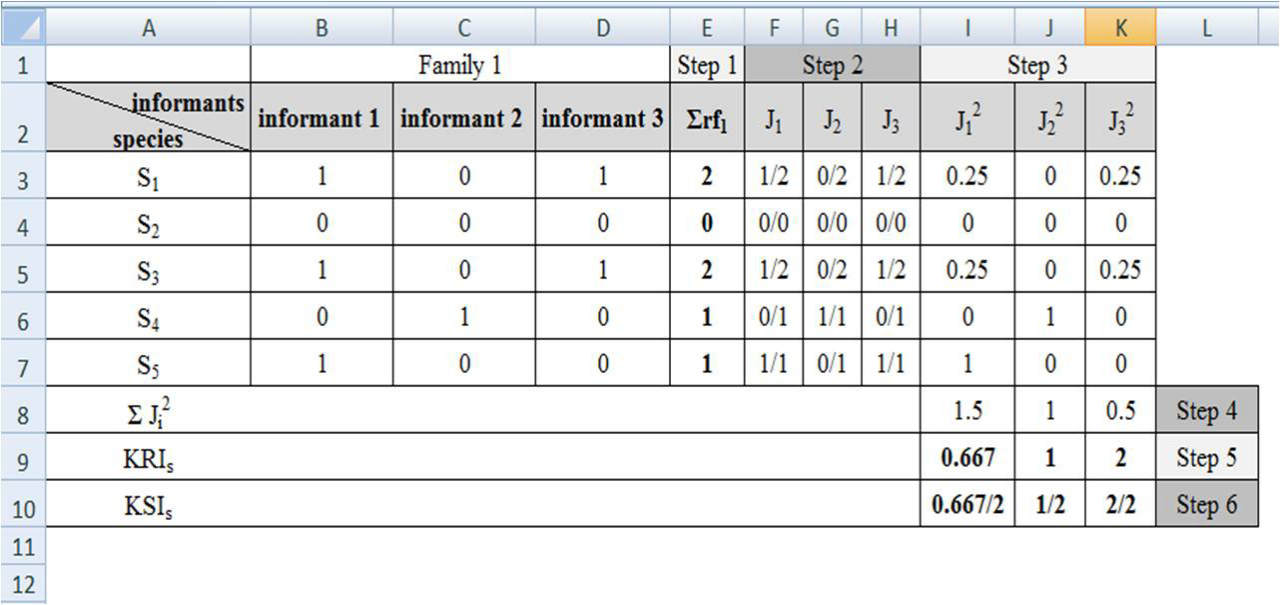
**Stages for calculating the KRI and KSI using the electronic spreadsheet software ^© ^Microsoft Office Excel 2007**.

To make these calculations easier and thus meet the assumptions of the proposal, we dictated that more than one person should be interviewed from each residence (when the sample unit is the family) and that a binary data matrix should be built (absence/presence) containing the record of a specific species (S_i_) cited by the informant (I_i_) (Figure 2). The steps in this process are as follows:

1. The sum of records of use (or cited) was calculated for the species (S_i_) in the family unit or community (f_i_): $\Sigma \text{R}{\text{f}}_{\text{i}}$ (Figure 2 - step 1);

2. J_i _was calculated as the ratio between the use of a species (S_i_) recorded by the informant (I_i_) and the sum of records of use for the species (S_i_) in the family or community (f_i_) (data obtained in step 1): ${\text{J}}_{\text{i}}={\text{R}}_{\text{i}}/\Sigma \text{R}{\text{f}}_{\text{i}}$ (Figure 2- step 2);

3. J_i_^2 ^was calculated for each species (S_i_) cited by the informant (I_i_) (Figure 2- step 3);

4. We carried out the sum of J_i_^2 ^values for all species (S_n_) cited by the informant (I_i_): ${\Sigma \text{J}}_{\text{i}}^{2}$ (Figure 2 - step 4);

5. Finally, we calculated the KRI as follows: $\text{KR}{\text{I}}_{\text{s}}=1/{\Sigma \text{J}}_{\text{i}}^{2}$

In this step, we note that the KRI is inversely proportional to the wealth of knowledge of the informant; in other words, the higher the J_i_^2 ^(wealth), the less the value of the KRI informant. The second index, KSI, is based on the ratio between the richness index of the informant and the maximum richness index of the family unit or community. It aims to evaluate the homogeneity of the knowledge.

The KSI is also a measure of distance, and the value may range from 0 to 1, with 1 being the value that expresses the lowest degree of sharing among a determined informant (KRI_i_) and the other components of the family unit or community (KRI_Max_) (Figure 2 - step 6).

$$\text{KSI}=\text{KR}{\text{I}}_{\text{i}}/\text{KR}{\text{I}}_{\text{Max}}.$$

Using the values for the KRI and KSI, the charting design (Figure 2) was developed to characterize the informants and sampling sufficiency. This chart illustrates how informants are distributed according to the richness and uniqueness of their knowledge of a particular cultural field. To this end, KRI and KSI values were transformed into log_10_, after which they were plotted in a Cartesian plane where the values of the KRI log_10 _were on the ordinate axis (x), and the KSI log_10 _values were on the abscissa axis (y).

### Data analysis

The normality of all scores obtained was checked with a Kolmogorov-Smirnov test. The Kruskal-Wallis non-parametric test [33] was used to compare the variance between each index's values in the categories for both variables. This test was chosen because all of the data were not normal and because samples of different sizes needed to be compared. The samples had different sizes because the intrafamily analysis was composed only of residences in which more than one person cited plants within the same category of use. Conversely, in the intracultural analysis, individuals who cited at least one species in a particular category were considered, making the number of residences and informants different among each category of use.

The Spearman correlation coefficient [33] was used to test the relationships among the values of the indices, the total number of known species and the number of unique species cited. In this test, the indices were calculated from the citations of specific plant use from participants who met our criteria of inclusion. Plants were considered unique in two areas: 1 - plants mentioned by a single person in the same household (intracultural analysis), 2 - plants mentioned only by one person in the community (intra-family analysis).

All statistical analyses were conducted with the BioEstat 5.0 program [34] considering a significance level of 95%.

A principal component analysis (PCA) was conducted using the software MVSP 3.1 [35] to check the tendencies in group formation as a function of the index values used in the correlation tests.

## Results

### Intrafamily variance

A total of 101 people were interviewed in 55 residences; however, according to the criteria of inclusion, only 85 interviews were analyzed (32 men and 53 women) from 36 residences. Families that did not show any interest in participating in the research and residences that included only one person did not meet the criteria of inclusion.

In the category of food plants, 91 ethnospecies were cited, with an average of 11.5 ethnospecies reported per residence. The smallest number of plants cited was recorded for the fuel category, with 48 ethnospecies and an average of 9.25 ethnospecies reported per residence. The medicinal category had the largest number of citations (159 ethnospecies), with an average citation of 27.2 ethnospecies per residence.

The smallest average KRI value was recorded for the medicinal category $\left(\bar{x}=0.2848\pm 0.55\right)$, suggesting that this category presents the greatest richness of knowledge among the informants of an individual residence. The food and fuel categories showed similar average KRI values $\left(\bar{x}=0.8511\pm 1.39\;\text{and}\;\bar{x}=8285\pm 1.611,\text{respectively}\right)$ and did not differ statistically from each other (*p *> 0.05); nevertheless, they were significantly different when compared to the medicinal category (*p *< 0.05) (Table 1).

**Table 1:** Statistical analysis of the knowledge richness index (KRI) and knowledge sharing index (KSI) intracultural for the three categories of use in a rural community of northeastern Brazil

	KRI		KSI	
**Categories** **of use**	**Mean ± SD**	**Mean ± SD**	**Number of** **people**	**Number of residences**

Fuel	0.8285 ± 1.611 a*	0.6198 ± 0.377 a	71	31

Food	0.8511 ± 1.39 a**	0.5769 ± 0.421 a	77	32

Medicinal	0.2848 ± 0.55 b	0.6644 ± 0.370 a	85	36

* H = 32.8168; *p *< 0.0001; ** H = 28.5688; *p *< 0.0001

Values followed by the same letters in the same column do not show significant differences

Concerning the results for KSI, the medicinal category showed a smaller overlap among family members on average$\left(\bar{x}=0.6644\pm 0.370\right)$. However, no difference was observed among the categories because the food and fuel categories behaved in a similar manner,$\left(\bar{x}=0.5769\pm 0.421\;\text{and}\;\bar{x}=0.6198\pm \text{respectively}\right)\text{s}0.377$, (Table 1).

These results suggested that the knowledge in this community about species richness is heterogeneous. Moreover, on average, slightly more than half of the local knowledge was not shared among individuals from the same residential unit. This lack of sharing did not significantly vary based on the analyzed category of use, given that the average values of KSI are greater than 0.5. For this reason, to obtain a greater richness of ethnobotanical data from families in this community, it would be necessary to interview the largest possible number of family members. This conclusion becomes more evident when analyzing the medicinal category because this category presented a high richness and low sharing of species information among family members.

As expected, the correlation tests among the total number of cited plants and the KRI were inverse and very significant (*p *< 0.001), and the strongest relationship was found for the medicinal category (rs = -0.9186; *p *< 0.0001). The fuel category presented the weakest relationship, although it was significant (Table 2).

**Table 2:** Correlation analysis between the knowledge richness index (KRI), the knowledge sharing index (KSI) and the number of plants cited (total and unique) per household (intracultural) for the three categories of use in a rural community in northeastern Brazil

	KRI		KSI	
**Categories of use**	**Rs**	**P**	**Rs**	**P**

**All plants**				

Fuel	-0.3842	0.0003	-0.1788	0.1016

Food	-0.6722	< 0.0001	-0.4206	< 0.0001

Medicinal	-0.9186	< 0.0001	-0.6057	< 0.0001

**Unique Plants**				

Fuel	-0.4645	< 0.0001	-0.2370	0.0289

Food	-0.7634	< 0.0001	-0.4924	< 0.0001

Medicinal	-0.9878	< 0.0001	-0.6540	< 0.0001

When comparing the KRI and KSI variables with the number of unique plants cited per residence, there was an increase in the value of the correlation coefficient among these variables, fluctuating between 7.69 and 20.9% for the KRI and between 8.3 and 35.2% for the KSI. These results show that the KRI and the KSI could represent information regarding the richness of local knowledge and unique records of known plants. This trend became more evident when the fuel category was analyzed because when the relationship between the KSI and the number of unique plants was tested, it began to show a significant correlation that was not previously registered.

Although the fuel category exhibited a weaker correlation, there was a general increase in the power of this type of analysis when only the uniquely cited records were employed (Table 2).

**Figure 3:**
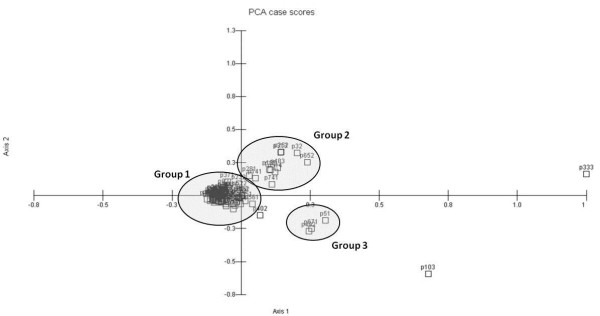
**Principal component analysis (PCA) of family units in a rural community in northeastern Brazil, considering the KRI and KSI values for three categories of use (fuel, food, and medicinal)**.

Based on a multivariate analysis, it was possible to identify the formation of three groups (Figure 3). Group 1 comprised of 81% of local people, and group 2 consists of 11 people. We observed that the formation of these group tended to correlate with information from the food category, which showed high KRI and KSI values; i.e., these informants showed a low richness of knowledge and sharing with the members of their families. Group 3 consists of only three informants. We found that all of the KRI and KSI values of the fuel category were equal for these informants, meaning that all of these informants know of few plants suitable for use as fuel, and that this knowledge is not commonly shared among members of their family.

### Intracultural variance

When analyzing food plants, 91 ethnospecies were cited, with an average of 5.8 ethnospecies reported per person. The smallest number of cited ethnospecies was recorded for the fuel category, with 48 ethnospecies and an average of 4.77 citations. The medicinal category showed the highest number of citations (159 ethnospecies), with an average citation of 14.8 ethnospecies per person.

Under this new configuration, the scores of the indices assume much higher (KRI) or much lower (KSI) values because the record of use for a specific species (S_i_) for each informant operates in the context of a much larger denominator. Given this assumption, the analysis should not only compare individuals' inter- and intrafamily relationships but also compare the relationships to the entire community.

We noted that previously indicated relationships (family units) were maintained, though with less power. On average, the KRI scores recorded were different among the categories of use (*p *< 0.05). The fuel category showed larger average values $\left(\bar{x}=256.184\pm \text{sd}513.855\right)$, followed by the food and medicinal categories $\left(\bar{\text{x}}=243.8861\pm \text{sd632}\text{.706;}\;\bar{x}=33.1315\pm \text{sd79}\text{.857}\right)$; $\bar{\text{x}}=33.1315\pm \text{sd}\;\text{79}\text{.857}$ (Table 3). Thus, the significance of the difference between the medicinal category and the other categories shows that knowledge of medicinal plants is much richer on average. The sharing of knowledge of medicinal plants was also very expressive overall, a result that was indicated by the lowest average KSI value recorded among the categories of use.

**Table 3:** Statistical analysis of the knowledge richness index (KRI) and knowledge sharing index (KSI) Intrafamily for the three categories of use in a rural community in northeastern Brazil

	KRI	KSI	
**Categories of use**	**Mean ± SD**	**Mean ± SD**	**N**

**Fuel**	256.184 ± 513.855a*	0.102 ± 0.205ab	76

**Food**	243.8861 ± 632.706b**	0.0806 ± 0.209a^#^	81

**Medicinal**	33.1315 ± 79.857c***	0.0742 ± 0.178b	85

*H = 5.456; *p *= 0.0195 **H = 29.208; *p *< 0.0001 ***H = 12.778; *p *= 0.0004; ^#^H = 4.773; *p *= 0.0289

Values followed by equal letters in the same column do not show significant differences

These data show that more knowledge is shared within the community than within family groups, especially in regards to the medical category. This finding suggests that obtaining the records of the plants used by the community would not require interviewing all of the community's members, given that knowledge is shared. Thus, if the researcher's target is the knowledge of the community, interviewing all family members (although interesting) would not be essential to documenting the community's knowledge.

The relationships among the total number of plants cited and the values of the indices were much weaker. They were found to be significant for the fuel and medicinal categories (Table 4). However, when comparing the KRI and KSI scores with the number of unique plants, the following increases were recorded: 105.64% for the fuel category, 195.11% for the medicinal category, and 300.37% for the food category.

**Table 4:** Correlation analysis between the knowledge richness index (KRI) and knowledge sharing index (KSI) and the number of cited plants per informant (intrafamily) for the three categories of use in a rural community in northeastern Brazil

		KRI		KSI	
**Categories of use**	**N of plants**	**Rs**	**P**	**rs**	**P**

**All plants**					

Fuel	48	-0.3082	0.0041	-0.3082	0.0041

Food	91	-0.1595	0.1447	-0.1595	0.1447

Medicinal	159	-0.2557	0.0181	-0.2557	0.0181

**Unique Plants**					

Fuel	16	-0.6338	< 0.0001	-0.6338	< 0.0001

Food	33	-0.6386	< 0.0001	-0.6386	< 0.0001

Medicinal	42	-0.7546	< 0.0001	-0.7546	< 0.0001

**Figure 4:**
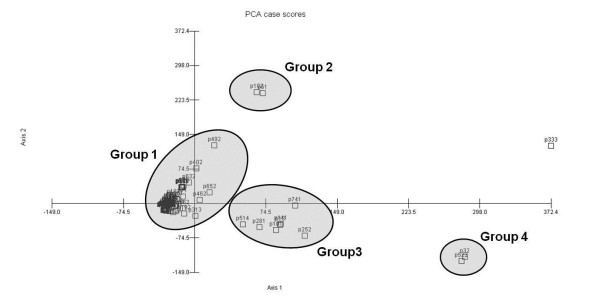
**Principal component analysis (PCA) among the informants of a rural community in northeastern Brazil, considering the KRI and KSI values for three categories of use (fuel, food and medicinal)**.

A multivariate analysis revealed the presence of four groups among the community's informants (Figure 4). Group 1 consists of 87.05% of the community. Group 2 consists of two people, and it was not possible to identify a trend within this group because the data from these two informants (scores of KRI and KSI for all categories of use) were very different from each other. For groups 3 and 4, it was possible to determine that knowledge of the food category influenced the formation of the groups. The members of this group are united by their low level of knowledge and sharing of this type of plant compared with the rest of the community, especially group 4, which showed lower values of KRI and KSI than group 3.

### Characterization and analysis of sample sufficiency

**Figure 5:**
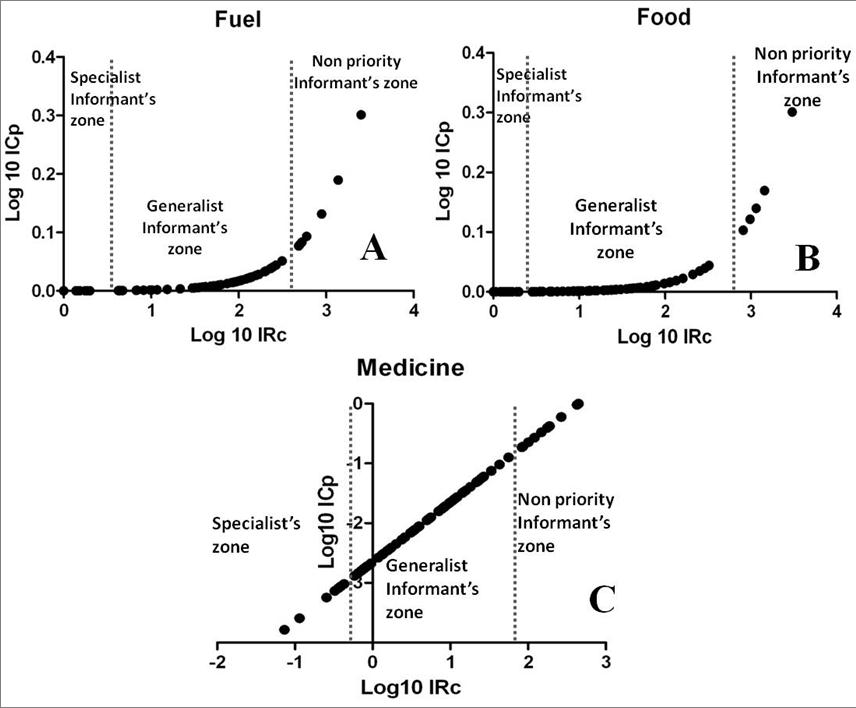
**Graphic representation of the expression (KRI; KSI) for the identification of groups and sample sufficiency of the three categories of use (A, B, and C)**.

Two basic trends were observed with respect to the distribution of the characteristics of informants. The first trend is present in the categories of fuel and food, in which most of the people aggregating and sharing characteristics related to the richness of their knowledge are observed on one side of the distribution. The other side of the distribution is characterized by people who could be removed from the analysis depending on the research objective (or who would not need to be interviewed) without causing significantly affecting the set of information that would be recorded, as they possess and share knowledge of few plants (Figure 5A and 5B).

The second trend is present in the medicinal category and supported by the formation of the two groups described above. Figure 5 illustrates the separation of a group of informants: the experts. These individuals are characterized by having the lowest scores in the indices, and there is a gap between them and the other informants (Figure 5C).

### Analysis of outliers

When the so-called "outliers" in the analysis were considered, a change in the position of the informants was observed for the food category, although it was not very expressive. This trend related to informants p341, p463, and p631, all of whom had their positions improve with the removal of the outliers from the analyses. Apparently, the total richness of cited plants helps define the positioning of informants when the outliers are removed from the analyses (Table 5).

**Table 5:** Comparison of the order of the first 12 positions of informants based on the presence and absence of outlier in calculating the KRI and KSI for the food category in a rural community in northeastern Brazil

Food				
**Informant**	**With outlier**	**Without outlier**	**Total cited plants**	**Unique plants**

p162	2°	1°	12	4

p372	4°	2°	13	2

p513	3°	3°	6	3

p261	5°	4°	17	2

p231	7°	5°	16	1

p282	6°	6°	13	2

p721	8°	7°	19	1

p341	15°	8°	15	0

p463	17°	9°	10	0

p662	9°	10°	6	1

p631	19°	11°	7	0

p251	10°	12°	5	1

In contrast to the food category, there was no change in the first positions of the medicinal category. These changes start to become expressive from the tenth position onward, highlighting the informants p261 and p21, who move from position 24 and 17 in the presence of the outliers to position 10 and 11 when the outliers are removed. Similar to the food category, richness seems to be more critical their position in the absence of the outliers than in their presence (Table 6).

**Table 6:** Comparison of the order of the first 12 positions of informants based on the presence and absence of outlier in calculating the KRI and KSI for the medicinal category in a rural community in northeastern Brazil

Medicinal				
**Informant**	**With outlier**	**Without outlier**	**Total cited plants**	**Unique plants**

p742	2°	1°	50	6

p631	3°	2°	37	3

p162	4°	3°	31	2

p721	5°	4°	34	2

p532	6°	5°	13	2

p512	7°	6°	31	2

p333	8°	7°	20	2

p231	9°	8°	32	1

p672	10°	9°	25	1

p261	24°	10°	19	0

p21	17°	11°	9	1

p331	11°	12°	15	1

## Discussion

The scores of the indices are good estimators of intra- and intracultural variation for the analyzed categories of use. Despite the focus of this analysis, there is nothing to prevent these indices from being tested and used as a sample unit for family groups. This unit would also consider other criteria and groupings implemented by the researcher (e.g., age range, gender and social function).

If the researcher intends to choose the group of informants that may be the target of their approach, it is possible to obtain a list of priorities that may augment their study through the addition of data offered by these indices. This information might be useful, for example, in situations in which the researcher has a very limited period of time in which to conduct a study. Applying this framework would make it possible to focus the researcher's efforts by identifying people who can contribute data relevant to the study's objectives, particularly in regard to the amount, or exclusivity, of information that they can provide.

A number of ethnobiology studies are based on intentional samplings [4,24,36-38], and as Tongco [39] noted, "methods in informant selection need to be actively discussed. Purposive sampling is a practical and efficient tool when used properly and can be just as effective as, and even more efficient than, random sampling". This approach, of course, depends on the objectives of the research being conducted. Our results show that the KRI and KSI values, as well as their charting designs, may assist in the intentional selection of informants.

Moreover, when information is retained by specific members of a community, it is advisable to use an intentional sample [39], which will save time, especially when information is not equally distributed and some potential informants may even be excluded. This type of selection could be applied when the parameter being studied is not shared by all of the members of a community [39].

Finally, we were able to evaluate how outliers interfere with these analyses and how other analyses may be conducted using these indices, such as determining the distance between the knowledge of a community compared to that of experts, as well as showing the importance of these individuals in retaining information of biological resources. There is no doubt that the results of this study have several implications and interpretations. For example, people identified as outliers by indices may have their information evaluated from different points of view, even if these individuals show a low richness of information and sharing. Plants that were rarely mentioned or shared may be: 1. introduced into the community's knowledge repository or 2. abandoned. In either outcome, the recovery and enhancement of such information becomes valuable from a cultural and scientific point of view.

A relevant aspect of our proposal is the objective of providing a simple tool for accessing a sampling effort using a procedure that is similar to the methods traditionally used in ecology. In our case, the researcher can continue to collect data while his sampling effort is evaluated. Thus, our index allows us access to information about how unique or shared botanical knowledge arises among families, communities or other social groups.

## Competing interests

The authors declare that they have no competing interests.

## Authors' contributions

TASA, ALSA and UPA have made substantial contributions to the conception and design analysis of the study and to the interpretation of the data. TASA, ALSA and JGM are responsible for the acquisition of the study data. All authors were involved in the drafting and revising of the manuscript and approved the final version.
